# Supplementary material for: LPC 18:2-Driven Apoptosis In Neutrophils Is Non-Inflammatory and Lipid Raft Dependent
Source: bioRxiv. 2025 Dec 12:2025.12.09.693266. Preprint. [Version 1] doi: 10.64898/2025.12.09.693266 (PMC12712933; doi:10.64898/2025.12.09.693266)

Supplementary Figure 1

A. Caspase Assay (Figure 1b, 3a, and 4c)

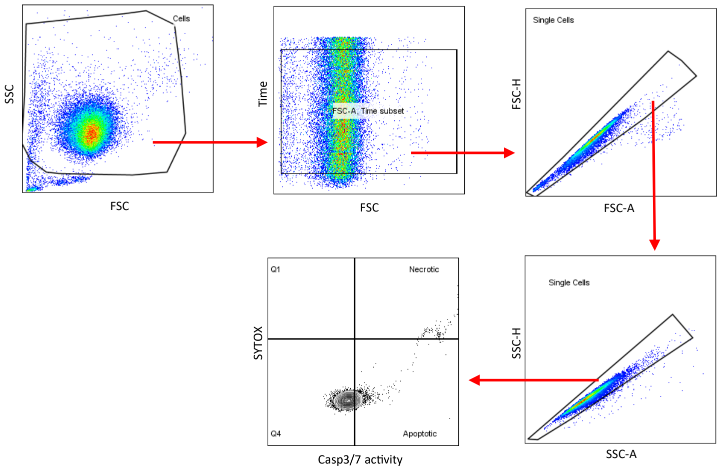

B. ROS Assay (Figure 1f and 4a)

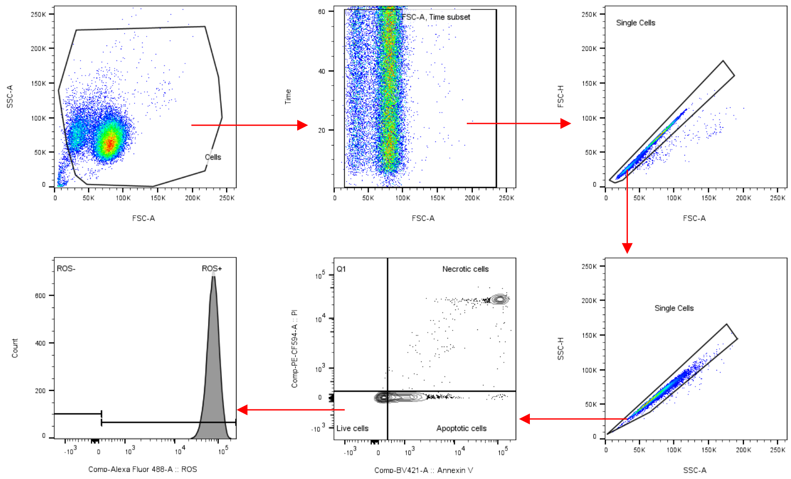

C. Cytochrome C (Figure 3b)

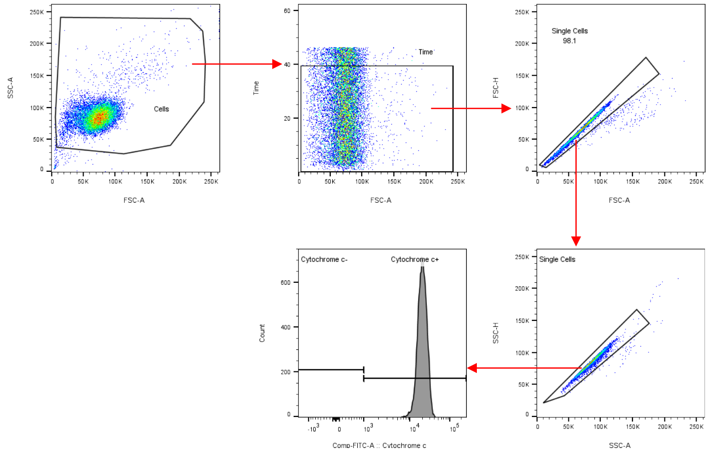

D. MitoTracker Assay (Figure 3c and 3d)

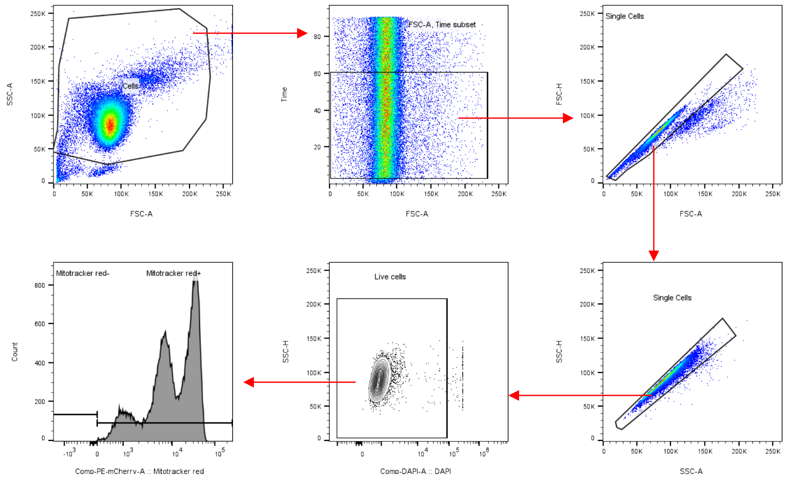

Supplement: Supplement 1 — Supplemental Figure 1. (A) Gating strategy for human neutrophil cleaved caspase-3/7 assay. (B) Gating strategy for human neutrophil ROS assay. (C) Gating strategy for human neutrophil cytochrome c release assay. (D) Gating strategy for human neutrophil MitoTracker assay. [file media-1.pdf]
